# Correction: *In situ* formation of transcriptional modulators using non-canonical DNA i-motifs

**DOI:** 10.1039/d6sc90123a

**Published:** 2026-06-10

**Authors:** Puja Saha, Deepanjan Panda, Diana Müller, Arunabha Maity, Harald Schwalbe, Jyotirmayee Dash

**Affiliations:** a School of Chemical Sciences, Indian Association for the Cultivation of Science Jadavpur Kolkata-700032 India ocjd@iacs.res.in; b Institute of Organic Chemistry and Chemical Biology, Center for Biomolecular Magnetic Resonance (BMRZ), Goethe University Max-von-Laue Strasse 7 Frankfurt D-60438 Germany

## Abstract

Correction for ‘*In situ* formation of transcriptional modulators using non-canonical DNA i-motifs’ by Puja Saha *et al.*, *Chem. Sci.*, 2020, **11**, 2058–2067, https://doi.org/10.1039/D0SC00514B.

The authors regret that an incorrect TEM image was inadvertently included in Fig. 2d. The corrected version of Fig. 2 is given below.

**Fig. 2:**
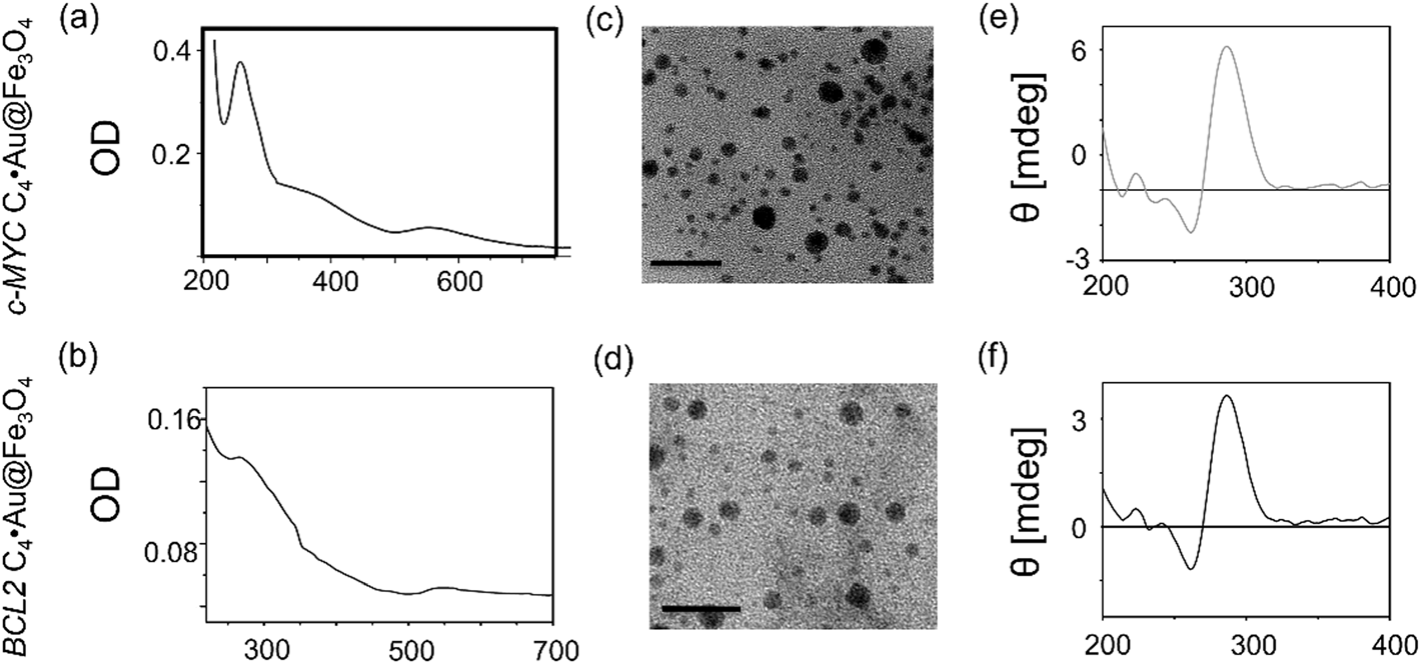
Characterization of i-motif DNA linked magnetic nanoparticles (C_4_·Au@Fe_3_O_4_ NPs). (a and b) UV-Vis absorption spectra of *c-MYC* C_4_·Au@Fe_3_O_4_ and *BCL2* C_4_·Au@Fe_3_O_4_ NPs at 25 °C; (c and d) TEM images of *c-MYC* and *BCL2* i-motif functionalized Au@Fe_3_O_4_ NPs (scale bar 50 nm); (e and f) CD spectra of *c-MYC* C_4_·Au@Fe_3_O_4_ and *BCL2* C_4_·Au@Fe_3_O_4_ NPs; buffer: 10 mM sodium cacodylate, pH 5.5.

The Royal Society of Chemistry apologises for these errors and any consequent inconvenience to authors and readers.

